# The esketamine-based multimodal low-opioid anaesthesia improves quality of recovery in elderly patients undergoing radical lung cancer surgery: a randomized controlled trial

**DOI:** 10.3389/fphar.2026.1769225

**Published:** 2026-05-28

**Authors:** Hai Zhou, Jiandong Wei, Zixuan Yang, Meiyan Zhou, Yifei Pan, Jiao Chen, Long Wang, Ying Ren, Yu Qi, Liwei Wang

**Affiliations:** 1 The Xuzhou Clinical College of Xuzhou Medical University, Xuzhou, Jiangsu, China; 2 Department of anesthesiology, Xuzhou Central Hospital, Xuzhou, Jiangsu, China; 3 Xuzhou Central Hospital, Southeast University, Xuzhou, Jiangsu, China

**Keywords:** elderly patients, esketamine, multimodal low-opioid anesthesia, quality of postoperative recovery, radical lung cancer surgery

## Abstract

**Background:**

The elderly population experiences diminished physiological function and reduced tolerance to surgery and anesthesia. This study explored the role of the esketamine-based multimodal low-opioid anaesthesia during the perioperative period in improving the quality of postoperative recovery in elderly patients undergoing thoracoscopic radical lung cancer resection.

**Materials and methods:**

This randomized controlled clinical trial enrolled 144 patients aged 60 years or older, with American Society of Anesthesiologists physical status grades II or III, undergoing elective thoracoscopic radical lung cancer resection. Participants were randomly assigned to the multimodal low-opioid strategy incorporating esketamine​ group (K Group)​ and the conventional opioid-based strategy group (C Group). The multimodal low-opioid strategy incorporating esketamine group received 0.5 mg/kg esketamine during anesthesia induction and 0.25 mg/kg/h for anesthesia maintenance. The conventional opioid-based strategy group received an equivalent volume of saline. Prior to thoracic closure, all patients received direct visualization-assisted intercostal nerve block corresponding to the surgical incision site as well as the adjacent upper and lower spaces. Postoperatively, the multimodal low-opioid strategy incorporating esketamine group received patient-controlled intravenous analgesia with 0.015 mg/kg/h esketamine and 0.015 ug/kg/h sufentanil, while the conventional opioid-based strategy group received 0.03 ug/kg/h sufentanil. The primary outcome was the Quality of Recovery Scale-15 (QoR-15) score on postoperative day 3. Secondary outcomes included QoR-15 score on postoperative day 1–2, postoperative NRS score at rest and movement, analgesic data, intraoperative hemodynamics, anesthetic drugs consumption, extubation time, PACU stay duration, and adverse events.

**Results:**

The multimodal low-opioid strategy incorporating esketamine group indicated significantly higher QoR-15 scores on postoperative day 3 compared to the conventional opioid-based strategy group (121 [117.75–124] vs. 115 [111.75–118.25], p < 0.001), primarily reflected in the physiological comfort, emotional state, and pain. Statistically significant differences in QoR-15 scores between the K and C groups were also observed on postoperative days 1 and 2 (P < 0.01). The multimodal low-opioid strategy incorporating esketamine group had significantly lower rest and dynamic NRS scores on postoperative days 1–3, fewer PCIA demands, a lower rescue analgesia rate, and less rescue analgesic consumption than the conventional opioid-based strategy group (all P < 0.05), indicating superior postoperative pain control. Compared with the conventional opioid-based strategy group, the multimodal low-opioid strategy incorporating esketamine group exhibited a significant reduction in perioperative opioid requirement (sufentanil plus remifentanil: 25 ± 4.62 ug vs. 38 ± 8.60 ug; 2.1 ± 0.35 mg vs. 2.5 ± 0.72 mg, p < 0.001) and propofol consumption (866.56 ± 76.44 mg vs. 968.36 ± 68.87 mg, p < 0.001). Esketamine provides dynamic and bidirectional regulation of blood pressure throughout different surgical phases in elderly patients undergoing radical lung cancer resection. Apart from a significantly lower incidence of postoperative nausea and vomiting in the multimodal low-opioid strategy incorporating esketamine group (10.0% vs. 21.4%, p < 0.05), the two groups showed comparable rates of other adverse effects, such as dizziness, headache, drowsiness, and restlessness (p > 0.05).

**Conclusion:**

The results of this study indicate that esketamine-based multimodal low-opioid anaesthesia effectively improves the quality of postoperative recovery in elderly patients undergoing thoracoscopic radical lung cancer resection (www.chictr.org.cn, registration number: ChiCTR2100051000).

## 1 Introduction

Following the COVID-19 pandemic, the widespread adoption of low-dose computed tomography (LDCT) has significantly increased the early diagnosis and surgical intervention rates for lung cancer, thereby reducing mortality from the disease (Leiter et al., 2023). According to the Global Cancer Statistics 2020, lung cancer ranks as the second most commonly diagnosed cancer worldwide after breast cancer, accounting for approximately 11.4% of all cancer diagnoses (Sung et al., 2021).

Video-assisted thoracoscopic surgery (VATS) is preferred over open surgery for radical lung cancer resection due to its benefits of fewer complications, improved quality of life, and better tolerance to adjuvant therapy (Klapper and D’Amico, 2015; Bendixen et al., 2016). However, within the framework of Enhanced Recovery After Surgery (ERAS), effectively accelerating recovery after VATS has remained a key focus for clinicians. This is particularly true for elderly patients with compromised physical function, as high-dose perioperative opioids can lead to various complications that hinder recovery (Zhang et al., 2025).

Esketamine is an NMDA receptor antagonist that inhibits NMDA receptors while promoting the release of endogenous opioids, acting by agonizing μ, δ, and κ opioid receptors (McMillan and Muthukumaraswamy, 2020; Johnston et al., 2023). It effectively alleviates postoperative pain, reduces opioid tolerance, and prevents hyperalgesia (Luginbühl et al., 2003). Additionally, it effectively relieves perioperative depression and cognitive impairment (Luscher et al., 2023; Wang et al., 2024), lowers the incidence of nausea and vomiting (Qi et al., 2024), improves early subjective QoR scores (Hung et al., 2024), and exhibits a lower incidence of psychiatric adverse reactions at equivalent doses, making it a relatively safe and reliable medication.

Despite this promising profile, high-quality evidence on the optimal administration regimen of esketamine for optimizing recovery in elderly lung cancer patients remains scarce. Given the accelerating population aging and increasing healthcare demands among the elderly, this trial specifically selected an elderly cohort as its research subjects.

Given that the Quality of Recovery-15 (QoR-15) is a comprehensive measure of postoperative health, it represents both a crucial recovery metric and an ideal indicator for quantifying esketamine’s potential benefits in analgesia, mood, and overall comfort (Myles et al., 2016). In this trial, continuous esketamine infusion was administered up to 48 h postoperatively. The QoR-15 score on postoperative day 3 (POD3) was selected as the primary outcome to capture recovery status after the acute pharmacological effects subsided, thereby providing a more objective assessment. This study is designed to investigate whether esketamine-based multimodal low-opioid anaesthesia enhances the quality of recovery in elderly patients undergoing VATS lung cancer resection.

## 2 Materials and methods

### 2.1 Study design and participants

This trial is a single-center, prospective, randomized controlled clinical trial approved by the Institutional Ethics Committee of Xuzhou Central Hospital (XZXY-LJ-20210331-044) and registered with the Chinese Clinical Trial Registry (ChiCTR2100051000). All subjects signed informed consent forms prior to enrollment. The study process adheres to the ethical principles outlined in the Declaration of Helsinki and the guidelines of the Consolidated Standards of Reporting Trials (CONSORT).

Between September 2022 and July 2023, 144 elderly patients scheduled for elective thoracoscopic radical resection of lung cancer at Xuzhou Central Hospital were recruited. Participants were randomly assigned to the low-opioid strategy group (K Group) and the conventional opioid-based strategy group (C Group). Inclusion criteria: (1) Scheduled for elective thoracoscopic radical resection of lung cancer; (2) American Society of Anesthesiologists (ASA) physical status grades II or III; (3) Patients aged ≥60 years old; (4) Agreement to use patient-controlled intravenous analgesia (PCIA); (5) Provision of written informed consent. Exclusion criteria: (1) Poorly controlled comorbidities (e.g., hypertension, diabetes, coronary heart disease, or cerebrovascular disease); (2) History of glaucoma or hyperthyroidism; (3) Known allergy to esketamine or other anesthetic agents used in the study; (4) Cognitive impairment or inability to communicate effectively.

### 2.2 Randomization and blinding

Participants were randomly assigned to the K group or the C group in a 1:1 ratio using a computer-generated random sequence. The allocation was concealed in sealed, opaque, sequentially numbered envelopes. An independent investigator opened the envelopes before anesthesia induction to disclose group assignments to the attending anesthesiologist. Study medications (esketamine or volume-matched saline placebo) were prepared by an independent nurse not involved in subsequent care according to the randomization list. The anesthesiologist administered these pre-prepared medications. To prevent assessment bias, the anesthesiologist was not involved in any postoperative data collection. Patients, surgeons, all postoperative care staff, and outcome assessors were fully blinded to group allocation. PCIA pumps were covered with opaque bags to maintain blinding throughout the postoperative period.

### 2.3 Anesthesia and monitoring

Both groups received preoperative NSAIDs for baseline analgesia and underwent standard fasting protocols. In the operating room, standard monitoring was established, including electrocardiogram, pulse oximetry, invasive arterial blood pressure, body temperature and the surgical pleth index.

The conventional opioid-based strategy group (C group): Patients in the C group received a standardized balanced general anesthesia protocol. Anesthesia was induced with midazolam (0.05 mg/kg), etomidate (0.15–0.2 mg/kg), sufentanil (0.3–0.6 ug/kg), and cisatracurium besylate (0.15–0.2 mg/kg). Immediately after induction, they received an intravenous bolus of normal saline equivalent in volume to the esketamine bolus used in the intervention group. Maintenance of anesthesia was achieved with continuous intravenous infusions of propofol (4–12 mg/kg/h) and remifentanil (0.1–1.0 μg/kg/min), supplemented with cisatracurium besylate as needed. A continuous infusion of normal saline, volume-matched to the esketamine infusion in the K group, was administered throughout surgery. Intraoperatively, drug dosages were titrated based on hemodynamic fluctuations and surgical requirements, with vasoactive agents administered as needed. Twenty minutes before the anticipated end of surgery, all infusions except propofol and remifentanil were stopped, and 0.1 mg/kg hydrocodone was administered intravenously for postoperative analgesia. Postoperative analgesia was provided via a PCIA pump containing sufentanil (0.03 μg/kg/h) and ondansetron (10 mg) in 100 mL normal saline, running at a continuous rate of 2 mL/h for 48 h, with a bolus option of 0.5 mL and a 15-min lockout interval. A single press provided an additional dose of 1 mL, with a lockout time of 15 min.

The multimodal low-opioid strategy incorporating esketamine group (K group): The anesthetic management for the K group was identical to that described for the C group, with the following exceptions: 1) Immediately after induction, patients received an intravenous bolus of 0.5 mg/kg esketamine instead of normal saline. 2)A continuous intraoperative infusion of esketamine at 0.25 mg/kg/h replaced the volume-matched normal saline infusion. 3)The postoperative PCIA solution contained esketamine (0.015 mg/kg/h) plus sufentanil (0.015 μg/kg/h) and ondansetron, instead of the higher-dose sufentanil regimen used in the C group.

Shared Procedures for Both groups: After 5 min of preoxygenation following induction of anesthesia, a double-lumen bronchial intubation was performed once hemodynamics stabilized. Its correct position was verified by auscultation and fiberoptic bronchoscopy. Subsequently, lung-protective mechanical ventilation was commenced, maintaining an end-tidal carbon dioxide pressure of 35–45 mmHg. Prior to thoracic closure, the surgeon performed intercostal nerve blocks under direct thoracoscopic visualization. A total of 5 mL of 0.5% ropivacaine was injected into each of the intercostal spaces corresponding to the incision site and the adjacent spaces above and below, with a total volume not exceeding 40 mL. Patients were transferred to the post-anesthesia care unit (PACU) with the endotracheal tube in place. The tube was removed upon meeting standard extubation criteria. Rescue analgesia with 50 mg of flurbiprofen axetil was administered if the rest Numeric Rating Scale (NRS) pain score was ≥4, with a maximum daily dose not exceeding 200 mg.

### 2.4 Outcome measurements

The primary outcome was the quality of recovery on postoperative day 3, evaluated using the Quality of Recovery Scale-15 (QoR-15) (Stark et al., 2013). This scale consists of 15 items, each scored from 0 to 10, encompassing five dimensions: physical comfort (5 items), physical independence (2 items), psychological support (2 items), emotional state (4 items), and pain (2 items). The total score ranges from 0 to 150, with higher scores indicating better recovery. The Chinese version, translated and validated by West China Hospital of Sichuan University with official permission for use, was employed (Bu et al., 2016) (Supplementary Figure S1). The questionnaire was completed by the patient or, when necessary, with the assistance of a surgical ward nurse who was blinded to the group assignments.

Secondary outcomes encompassed a range of perioperative measures. These included QoR-15 score on POD1-2, the rest and dynamic NRS scores on postoperative days 1–3, PCIA demand frequency, rescue analgesia rate, rescue analgesic consumption, intraoperative hemodynamic parameters (systolic, diastolic, and mean arterial pressure, and heart rate), consumption of anesthetics (propofol and opioids), as well as recovery metrics (extubation time and PACU stay duration). The incidence of postoperative adverse reactions, such as nausea and vomiting, dizziness, headache, nightmares, hallucinations, and somnolence, was also recorded. Hemodynamics were measured at seven key time points: before anesthesia induction (T0), after induction (T1), immediately after intubation (T2), 5 min after positioning (T3), at skin incision (T4), 1 h after surgery start (T5), and at surgery completion (T6).

### 2.5 Sample size calculation

Based on preliminary pre-experimental data, the Quality of Recovery-15 (QoR-15) scores for elderly patients undergoing VATS were 113.6 ± 10.37. Previous literature indicates that the minimum clinically important difference (MCID) for QoR-15 is 6 (Myles and Myles, 2021). This study employed PASS 2023 statistical software for sample size calculation, setting test power as 90% and the test level as 0.05. With a 1:1 sample size ratio between the group K and group C, calculations indicated 64 patients per group were required. Accounting for a 10% dropout rate, the final actual enrollment per group was 72 patients, totaling 144 patients.

### 2.6 Statistical analysis

All statistical analyses were performed on an intention-to-treat (ITT) principle using SPSS software (version 26.0, IBM Corp.) and GraphPad Prism (version 9.0, GraphPad Software). The normality of data distribution was assessed using the Shapiro-Wilk test. Continuous data are presented as mean ± standard deviation (SD) for normally distributed variables and as median (interquartile range, IQR) for non-normally distributed variables. Between-group comparisons for continuous data were conducted using the independent samples t-test for normally distributed data and the Mann-Whitney U test for non-normally distributed data. Categorical data are expressed as numbers (percentages) and were compared using the Chi-squared (χ^2^) test or Fisher’s exact test, as dictated by cell count assumptions.

The primary outcome was compared between groups using the independent samples t-test. A two-sided P-value <0.05 was considered statistically significant for the primary outcome. To control for the increased risk of Type I error, the Bonferroni correction was applied to the analysis of all secondary outcomes, where the threshold for significance was adjusted accordingly. To address the potential confounding effect of the observed baseline imbalance in vasopressor use, multivariable linear regression analyses were employed for key outcomes. The primary outcome (QoR-15 score on postoperative day 3) and secondary outcomes including intraoperative opioid consumption (sufentanil and remifentanil) and intraoperative hemodynamic variability indices (Coefficient of variation of mean arterial pressure and Coefficient of variation of mean heart rate) were treated as dependent variables in separate models. In each model, the treatment “Group” (intervention vs. control) was the independent variable of interest, and “Intraoperative Norepinephrine Use” (yes/no) was forced into the model as a covariate for adjustment. Results of these regression analyses are reported as unstandardized regression coefficients (B) with their 95% confidence intervals and corresponding P-values.

## 3 Results

**FIGURE 1:**
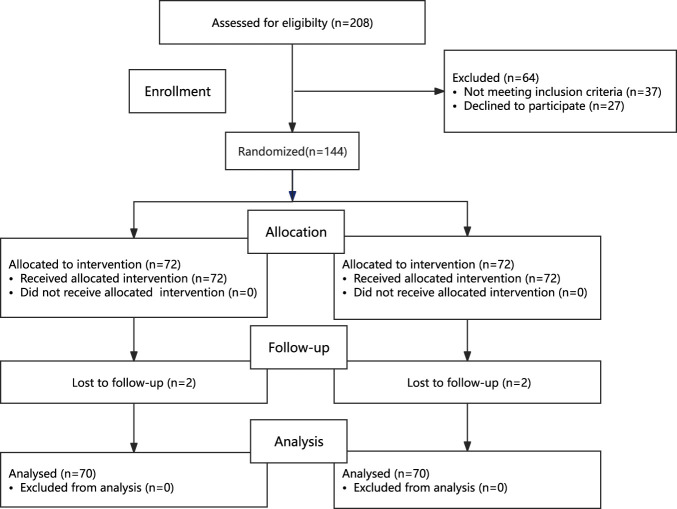
Flow diagram.

Between September 2022 and July 2023, 208 patients were assessed for eligibility, and 64 were excluded. The resulting 144 eligible patients were randomly allocated to the multimodal low-opioid strategy incorporating esketamine group or the conventional opioid-based strategy group. All randomized patients underwent the intended surgical procedure under the designated anesthesia regimen. During follow-up, two patients in each group were lost, which yielded 140 patients who were retained for analysis (70 in each group). The trial profile is summarized in Figure 1. As shown in Table 1, the groups were balanced on all baseline and surgical variables except for epinephrine usage, for which a significant intergroup difference was observed (P < 0.01) (Table 1).

**TABLE 1:** Baseline characteristics and surgical data.

Characteristic	Patients, no. (%)		P value
	K group (n = 70)	C group (n = 70)	
Age, mean ± SD, y	67.41 ± 3.38	66.96 ± 3.74	0.449
Sex	​	​	0.310
Female	36 (51.4)	30 (42.9)	​
Male	34 (48.6)	40 (57.1)	​
BMI, mean ± SD, kg/m2	25.00 ± 3.87	26.02 ± 2.83	0.074
Current smoker	22 (31.4)	19 (27.1)	0.577
Preoperative examination			
Hb (g/dL)	133.16 ± 14.94	136.39 ± 12.54	0.168
RBC(×10^12^/L)	4.30 ± 0.44	4.45 ± 0.51	0.73
WBC (×10^9^/L)	6.16 ± 1.41	6.06 ± 1.93	0.73
PLT (×10^9^/L)	245.51 ± 85.51	239.37 ± 75.41	0.653
NE (×10^9^/L)	3.70 ± 1.37	4.00 ± 1.33	0.2
ASA physical status	​	​	0.753
II	65 (92.9)	64 (91.4)	​
III	5 (7.1)	6 (8.6)	​
Comorbidities			
Hypertension	17 (24.3)	21 (30.0)	0.447
Diabetes	11 (15.7)	13 (18.6)	0.654
Cardiovascular disease	5 (7.1)	7 (10.0)	0.645
COPD	2 (2.9)	3 (4.3)	0.649
Cerebral infarction	4 (5.7)	6 (8.6)	0.512
Preoperative QoR-15 value	143 (138–144)	141 (139–143)	0.161
Length of preoperative hospital stay, median M (P25, P75), day	4 (3–5)	4 (2–4)	0.72
Duration of operation, mean ± SD, min	151 ± 27.05	145 ± 27.49	0.206
Duration of anesthesia, mean ± SD, min	172 ± 26.25	166 ± 29.54	0.228
Bleeding loss, mean ± SD, mL	108 ± 54.58	116 ± 36.70	0.311
Urine volume, mean ± SD, mL	431.86 ± 137.32	464.71 ± 128.03	0.145
Crystalloid infusion volume, mean ± SD, mL	665.71 ± 156.63	691.43 ± 167.02	0.349
Colloidal fluid infusion volume, mean ± SD, mL	389.29 ± 78.89	407.14 ± 85.67	0.202
Norepinephrine	9 (12.9)	28 (40)	<0.01

Abbreviations: ASA, american society of anesthesiologists physiological status; SD, standarddeviation; Hb, Hemoglobin; RBC, red blood cell; WBC: leukocyte; PLT, platelet; NE, neutrophilicgranulocyte.

### 3.1 QoR-15 scores

As shown in Table 2, the K group demonstrated a significantly higher QoR-15 score on postoperative day 3 compared to the C group (121 [117.75–124] vs. 115 [110–118.25], P < 0.001), representing a clinically meaningful improvement in recovery quality.

**TABLE 2:** Primary outcomes.

Outcome measures	K group (n = 70)	C group (n = 70)	P value
POD-3 QoR-15 scores	121 (117.75–124)	115 (111.75–118.25)	<0.001
Physical comfort	40 (39–41)	38 (37–40)	<0.001
Physical independence	11 (10–12)	11 (10–11)	0.375
Psychologic support	20 (19–20)	20 (18–20)	0.103
Emotional state	32 (30–33)	29 (27.75–31)	<0.001
Pain	18 (18–20)	18 (16.75–19)	<0.001

A subscale analysis of the QoR-15 on postoperative day 3 revealed that the K group had significantly higher scores than the C group in the dimensions of physical comfort (40 [39–41] vs. 38 [37–40]; P < 0.001), emotional state (32 [30–33] vs. 29 [27–31]; P < 0.001), and pain (18 [18–20] vs. 18 [16–19]; P < 0.001), as detailed in Table 2.

Statistically significant differences in QoR-15 scores between the K and C groups were also observed on postoperative days 1 and 2 (P < 0.01), although these differences did not reach the minimal clinically important difference (MCID) (Table 3).

**TABLE 3:** QoR-15 scores on POD1-2.

Outcome measures	K group (n = 70)	C group (n = 70)	P value
POD-1			
QoR-15 scores	106 (102.75–108)	103 (101–105)	0.002
Physical comfort	36 (34.75–37)	35 (34–36)	0.014
Physical independence	7 (6–7)	7 (6–8)	0.326
Psychologic support	19.5 (18.75–20)	20 (18–20)	0.868
Emotional state	27 (25–27)	26 (25–27)	0.010
Pain	17 (16–17)	17 (16–17)	<0.001
POD-2			
QoR-15 scores	110 (107–113)	107 (105–109)	<0.001
Physical comfort	37 (36–39)	36 (35–37.25)	0.003
Physical independence	8 (8–9)	8 (7–9)	0.275
Psychologic support	20 (19–20)	20 (18–20)	0.569
Emotional state	28 (26.75–30)	27 (26–28)	0.005
Pain	17 (16.75–18)	17 (16–17)	0.009

### 3.2 Postoperative NRS score at rest and movement and analgesic data

As shown in Table 4, the K group exhibited significantly lower rest and dynamic NRS pain scores compared with the C group on postoperative days 1–3 (P < 0.05 for all). Specifically, on postoperative day 3, the rest NRS score in the K group was 2 (2–2) versus 2 (2–3) in the C group (P = 0.02), and the dynamic NRS score was 3 (3–4) versus 4 (3–4) in the C group (P = 0.006), indicating superior pain control throughout the early postoperative period.

**TABLE 4:** Postoperative NRS score and Analgesic data.

Outcome measures	K group (n = 70)	C group (n = 70)	P value
NRS score at rest			
POD-1	3 (3–3)	3 (3–4)	<0.001
POD-2	3 (2–3)	3 (3–3)	0.01
POD-3	2 (2–2)	2 (2–3)	0.006
NRS score on movement			
POD-1	4 (4–5)	5 (4–5)	0.013
POD-2	4 (4–4)	4 (4–5)	<0.001
POD-3	3 (3–4)	4 (3–4)	0.02
PCIA demand frequency	12.5 (9–15)	15 (12–17)	0.002
Rescue analgesia, n (%)	10 (14.3)	23 (32.9)	0.01
Rescue analgesic consumption per patient (mg)	0 (0–0)	0 (0–100)	0.008

In terms of analgesic requirements, the K group had significantly fewer PCIA demand frequencies (12.5 [9–15] vs. 15 [12–17], P = 0.002), a lower rescue analgesia rate (14.3% vs. 32.9%, P = 0.01), and lower rescue analgesic consumption per patient (0 [0–0] mg vs. 0 [0–100] mg, P = 0.008) compared with the C group. These findings suggest that the esketamine-based regimen not only improved postoperative pain relief but also reduced the need for additional analgesic interventions, which may contribute to enhanced patient comfort and recovery.

### 3.3 Hemodynamic characteristics

**FIGURE 2:**
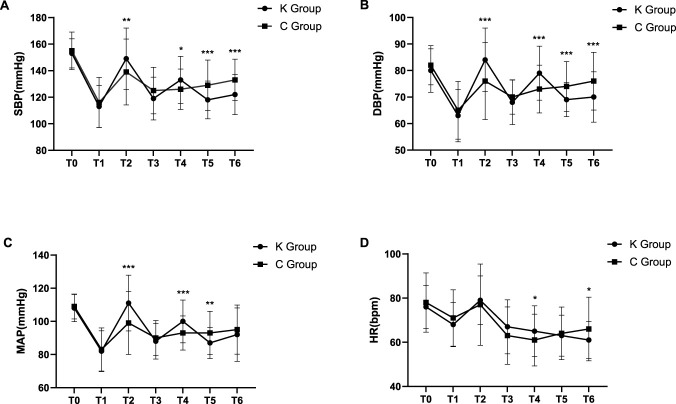
Comparison of intraoperative hemodynamic changes **(A)** Systolic Blood Pressure, **(B)** Diastolic Blood Pressure, **(C)** Mean Arterial Pressure, **(D)** Heart Rate) between the multimodal low-opioid strategy incorporating esketamine group and the conventional opioid-based strategy group.

Compared to baseline, blood pressure and heart rate decreased after anesthesia induction in both groups. Relative to the C group, the K group showed significantly higher SBP, DBP, and MAP at time points T2 and T4, as well as a higher HR at T4 (P < 0.05). Conversely, the K group had significantly lower SBP and DBP at T5 and T6, a lower MAP at T5, and a lower HR at T6 (P < 0.05) (Figure 2).

### 3.4 Consumption of anesthetics

**FIGURE 3:**
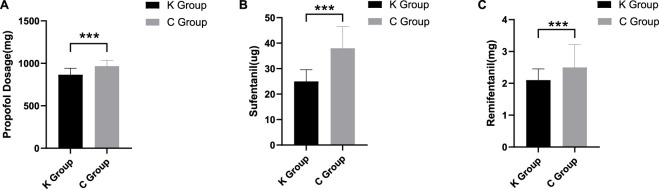
Comparison of perioperative propofol **(A)**, sufentanil **(B)** and remifentanil **(C)** use between the multimodal low-opioid strategy incorporating esketamine group and the conventional opioid-based strategy group.

Notably, the consumption of perioperative anesthetics was significantly lower in the K group than in the C group. This included reduced usage of propofol (866.56 ± 76.44 mg vs. 968.36 ± 68.87 mg, P < 0.05) (Figure 3A), sufentanil (25 ± 4.62 ug vs. 38 ± 8.60 ug, P < 0.001) (Figure 3B), and remifentanil (2.1 ± 0.35 mg vs. 2.5 ± 0.72 mg, P < 0.001) (Figure 3C).

### 3.5 Recovery metrics

**FIGURE 4:**
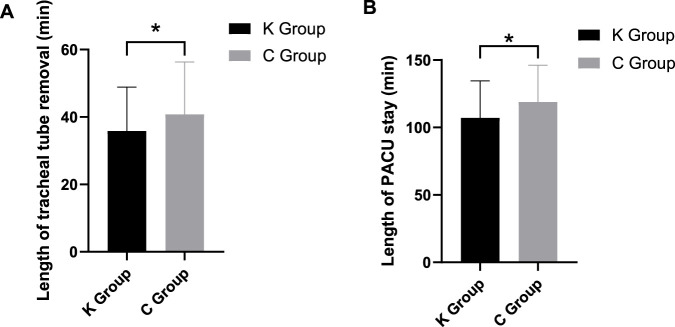
Comparison of Length of tracheal tube removal **(A)** and Length of PACU stay **(B)** between the multimodal low-opioid strategy incorporating esketamine group and the conventional opioid-based strategy group.

During the anesthesia recovery phase, both the extubation time (35.8 ± 13.02 min vs. 40.8 ± 15.5 min, P < 0.05; Figure 4A) and the PACU stay duration (107.2 ± 27.4 min vs. 118.9 ± 27.2 min, P < 0.05; Figure 4B) were significantly shorter in the K group than in the C group.

### 3.6 Incidence of adverse events

The assessment of adverse reactions revealed that the incidence of nausea and vomiting was significantly lower in the K group than in the C group (10.0% vs. 21.4%, P < 0.05). No other significant differences were observed between the two groups in the incidence of dizziness, headache, drowsiness, or restlessness (P > 0.05) (Table 5).

**TABLE 5:** Adverse event.

Outcome measures	K group (Number)	C group (Number)
Nausea and vomiting	7	15
Dizziness	1	2
Headache	2	3
Nightmares	2	2
Hallucination	0	0
Hypersomnia	1	1
Dysphoria	0	1
Pruritus	1	2
Respiratory depression	2	3
Arrhythmia	0	1
Delirium	1	0
Infection	2	2
Pneumothorax	1	1
Bronchopleural fistula	0	1
Pulmonary embolism	0	0

### 3.7 Multivariable regression adjustment

Multivariable linear regression analyses were performed to assess the independent association of the esketamine-based multimodal low-opioid strategy with key outcomes after adjusting for the baseline imbalance in intraoperative norepinephrine use. After forcing “Intraoperative Norepinephrine Use” (yes or no) into the models as a covariate, the treatment “Group” (intervention vs. control) remained a highly significant independent predictor.

Specifically, the intervention was associated with a significantly higher QoR-15 score on postoperative day 3 (B = 5.52, 95% CI 3.97 to 7.06, P < 0.001), and with significantly lower total consumption of both sufentanil (B = −12.61, 95% CI -15.02 to −10.19, P < 0.001) and remifentanil (B = −0.39, 95% CI -0.58 to −0.19, P < 0.001). Concerning intraoperative hemodynamics, the intervention was independently associated with a lower heart rate variability (B = −0.027, 95% CI -0.042 to −0.012, P < 0.001) but a slightly higher mean arterial pressure variability (B = 0.018, 95% CI 0.001 to 0.034, P = 0.033). The covariate “Intraoperative Norepinephrine Use”itself showed no significant association with any of these outcomes in the models (all P > 0.70) (Supplementary Table S1).

## 4 Discussion

Our findings indicate that a multimodal low-opioid anaesthesia strategy incorporating esketamine provides multimodal benefits, leading to a significantly enhanced quality of recovery by postoperative day 3 in elderly lung cancer surgery patients. This enhancement is evidenced by marked improvements across key domains such as physical comfort, emotional state, and pain control.

Results showed that on postoperative days 1–2, QoR-15 scores remained relatively low in both groups. The between-group differences in QoR-15 scores were statistically significant but smaller in magnitude (3 points), not reaching the MCID. This may be attributed to the complex stress state induced by surgical trauma and thoracic tube stimulation (Kehlet and Dahl, 2003), during which the effects of esketamine had not yet fully manifested. Additionally, residual effects of anesthetic agents may have influenced patients' subjective perceptions (Apfel et al., 2012). A statistically significant, six-point higher median QoR-15 score was observed in the K group compared to the C group on POD3, which meets the threshold for the established minimal clinically important difference (MCID) of six points for the QoR-15 scale (Myles and Myles, 2021). Therefore, our results suggest that the esketamine-based multimodal regimen may provide a clinically meaningful improvement in early recovery quality after thoracoscopic surgery. Specifically, this clinically important difference at POD3 likely reflects the cumulative effect of the sustained multi-modal analgesia regimen, a core component of the esketamine-based low-opioid strategy, which included modified postoperative PCIA settings. Therefore, the recovery benefit at POD3 should be interpreted as the result of the integrated perioperative strategy, rather than attributed solely to the intraoperative pharmacologic effect of esketamine. However, it is important to acknowledge the borderline nature of this effect, as the point estimate equals the MCID. Future studies with larger sample sizes are warranted to confirm the magnitude and consistency of this benefit. Overall, this pattern indicates that the beneficial effect of the intervention was detectable early in the recovery process and increased progressively over time, culminating in a difference that achieved clinical relevance by POD3.

Specifically, the K group presented significantly lower rest and dynamic NRS scores on postoperative days 1–3, as well as fewer PCIA demands, lower rescue analgesia rate and consumption compared with the C group (all P < 0.05). These objective analgesic indicators confirmed that the esketamine-based multimodal regimen provided effective and sustained pain relief, which laid a solid foundation for the better recovery outcomes. The underlying mechanism may involve that esketamine continuously modulates central nervous system function: by antagonizing NMDA receptors, it inhibits hyperalgesia and central sensitization, thereby alleviating postoperative pain (Luginbühl et al., 2003); By antagonizing NMDA receptors and reducing glutamate excitotoxicity, it restores impaired neural plasticity in states of anxiety (McIntyre et al., 2021); simultaneously, by inhibiting M1 polarization of microglia and modulating the BDNF-TrkB pathway associated with preoperative sleep disturbances (Wen et al., 2024), it further improves recovery across dimensions such as physical comfort, emotional state, and pain, ultimately leading to a significant increase in QoR-15 scores. Additionally, esketamine undergoes hepatic metabolism via microsomal enzymes to form norketamine. This active metabolite exhibits significant pharmacological activity, with an analgesic effect equivalent to 1/5–1/3 of esketamine’s potency and a longer elimination half-life, which may contribute to the sustained therapeutic effects (Kamp et al., 2020).

As designed, this multimodal low-opioid anesthetic regimen resulted in lower perioperative opioid consumption and associated adverse effects, including nausea and vomiting, intestinal paralysis, respiratory depression, and hyperalgesia (Mercadante et al., 2019; Baldo, 2021). Consistent with this principle, the incidence of nausea and vomiting was significantly lower in the K group than in the C group, confirming that an esketamine-based multimodal low-opioid regimen enhances patient comfort (Feng et al., 2024; Qi et al., 2024). The underlying mechanism may be attributed to esketamine’s unique profile as an NMDA receptor antagonist, which provides sedation and analgesia without excessive central nervous system depression. This helps alleviate postoperative anxiety and stress-induced pain, thereby improving physical comfort and overall recovery quality (Wang et al., 2021; Cheng et al., 2022).

Research findings indicate that the esketamine-based multimodal low-opioid strategy exerts a complex influence on hemodynamics. At high-stimulus phases, blood pressure was higher in the K group, consistent with esketamine’s sympathomimetic properties which may augment stability under acute stress (Takki et al., 1972; Kamp et al., 2021). In contrast, blood pressure was lower in the K group during later, sustained surgical phases. The potent analgesic and anti-stress effects of the strategy likely mitigated the surgical stress response, potentially reducing the need for supportive vasoactive medications (Wang et al., 2021). Crucially, to isolate the effect of the strategy from the baseline imbalance in vasopressor use, we performed multivariable regression adjusting for intraoperative norepinephrine administration. This analysis confirmed that the strategy was independently associated with a reduction in heart rate variability (B = −0.027, p < 0.001), suggesting enhanced hemodynamic stability in this domain. However, it was also associated with a slight increase in mean arterial pressure variability (B = 0.018, p = 0.033). This nuanced result suggests that the strategy’s overall hemodynamic profile is multifaceted: while promoting heart rate stability, its net effect on blood pressure may reflect a balance between its analgesic/stress-reducing properties and its inherent sympathomimetic activity. Therefore, the observed hemodynamic outcomes are best interpreted as the net effect of the integrated pharmacological strategy rather than a singular drug effect. Future studies integrating quantitative vasoactive drug data with advanced hemodynamic monitoring will help further elucidate these mechanisms.

The results of this study indicate that perioperative esketamine-based multimodal low-opioid anesthetic regimen significantly shortens the time to extubation and PACU stay compared to the standard opioid-based regimen in patients undergoing thoracoscopic lung cancer resection. This positive outcome primarily stems from a combination of the protocol design and esketamine’s pharmacology. As per the study protocol, the intervention group received lower doses of intraoperative and postoperative opioids and propofol, agents known to prolong emergence and respiratory recovery (Ma et al., 2023). Therefore, the reduction in these anesthetic agents was an inherent feature of the intervention strategy rather than an independently measured effect of esketamine.​ Additionally, the underlying mechanism may involve the activation of glutamatergic neurons in the paraventricular thalamic nucleus (PVT) (Duan et al., 2024), which promotes arousal by stimulating key neural pathways involved in consciousness recovery (Wang et al., 2023). Concurrently, esketamine provides sustained analgesia and mood stabilization (Luo et al., 2024), which minimizes pain, agitation, and related complications in the PACU, allowing patients to meet discharge criteria more rapidly. In summary, this study suggests that integrating esketamine into a multimodal low-opioid ERAS strategy can synergistically optimize recovery by reducing overall anesthetic burden and potentially leveraging esketamine’s unique properties, though the independent contribution of esketamine requires further blinded investigation.

Interpreting the findings of this study within the context of recent literature exploring the role of esketamine in geriatric thoracic surgery, we found that: While randomized controlled trials (RCTs) by Fu et al. (2025) and Zhan et al. (2025) overlap with the present study in patient population (elderly lung cancer surgery patients) and intervention drug (esketamine), key differences in research focus and design underscore the unique value of this work. Fu et al. specifically focused on high-risk patients with “cognitive frailty” with postoperative delirium (POD) as the primary outcome. Their findings showed that esketamine exerted neuroprotective effects in non-acute care patients compared to placebo, as reflected by reduced POD incidence and lower levels of inflammatory markers (IL-6, S100β). In contrast, Zhan et al. evaluated the impact of esketamine-based total opioid-free anesthesia on postoperative cognitive dysfunction (POCD). Together, these two studies confirm esketamine’s potential to mitigate specific neurocognitive complications in this cohort. The present study significantly expands existing knowledge in two key aspects. First, regarding research objectives and outcomes, it is the first to adopt the gold standard for patient-reported outcomes - the Quality of Recovery-15 (QoR-15) - to assess early overall recovery quality in elderly lung cancer patients undergoing esketamine-based multimodal low-opioid anesthesia. Our results show this regimen not only achieved a clinically meaningful minimum clinically important difference (MCID) in QoR-15 scores by postoperative day 3 but also improved multiple dimensions, including physical comfort, emotional state, and pain control. This extends esketamine’s benefits beyond “neuroprotection” to the broader clinical goal of “patient-centered comprehensive recovery,” providing new evidence for its use as a core agent in Enhanced Recovery After Surgery (ERAS) protocols. Second, in terms of clinical pathways, unlike Zhan et al.‘s aggressive “opioid-free” strategy, the present study validated a feasible “low-opioid” approach that is more consistent with routine clinical practice. This protocol significantly reduced perioperative opioid use and opioid-related adverse events, while allowing minimal opioid doses for intraoperative rescue analgesia. This finding has important practical value: it demonstrates that key clinical benefits - reduced opioid consumption and improved recovery - can be achieved without pursuing the theoretical “opioid-free” goal, offering clinicians a balanced option that prioritizes both safety and efficacy.

Despite clarifying the beneficial role of the multimodal low-opioid anaesthesia strategy incorporating esketamine in enhancing recovery quality after thoracoscopic lung cancer resection in elderly patients, this study has several limitations. First, the lack of therapeutic drug monitoring precludes the establishment of a precise dose-response relationship between esketamine plasma concentration and clinical effects, and obscures any potential correlation between pharmacokinetic fluctuations and adverse events. Secondly, the data collection for this trial was limited to the first 3 days postoperatively and did not track patients' long-term recovery outcomes, such as chronic pain. Consequently, it remains unclear whether esketamine effectively prevents chronic pain in patients undergoing thoracoscopic lung cancer resection. This limitation partially restricts the clinical applicability of the study’s conclusions. Finally, this trial was limited to a single type of surgical procedure and did not include patients undergoing open surgery. Future studies should further validate the therapeutic value of esketamine across different surgical techniques.

## 5 Conclusion

In summary, compared to a traditional opioid-based general anesthetic, an esketamine-based multimodal low-opioid anaesthesia regimen significantly enhances recovery quality on postoperative day 3 in elderly patients undergoing thoracoscopic radical lung cancer surgery.

## Data Availability

The raw data supporting the conclusions of this article will be made available by the authors, without undue reservation.
