# Supplementary material for: The esketamine-based multimodal low-opioid anaesthesia improves quality of recovery in elderly patients undergoing radical lung cancer surgery: a randomized controlled trial
Source: Front Pharmacol. 2026 May 28;17:1769225. doi: 10.3389/fphar.2026.1769225 (PMC13253306; doi:10.3389/fphar.2026.1769225)
Supplement: Supplementary file 1 [file Supplementaryfile1.docx]

**Supplementary Figure 1**. the Quality of Recovery-15 Scale—Chinese version (QoR-15C)

**QoR-15调查问卷**

日期： / / 研究编号：

术前：□ 术后：□

完成时间：

**A部分**

您在过去的24小时里感觉如何？

（选择0到10, 0表示从来没有[极差]，10表示一直都是[很好]）

1. 呼吸顺畅 从来
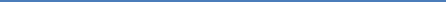
 一直

没有 0 1 2 3 4 5 6 7 8 9 10 都是

1. 食欲佳 从来
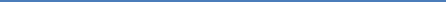
 一直

没有 0 1 2 3 4 5 6 7 8 9 10 都是

1. 感觉精力充沛

从来
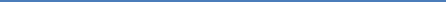
 一直

没有 0 1 2 3 4 5 6 7 8 9 10 都是

1. 睡眠质量佳 从来
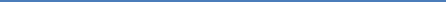
 一直

没有 0 1 2 3 4 5 6 7 8 9 10 都是

1. 独立完成个人卫生和上厕所

从来
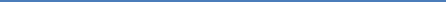
 一直

没有 0 1 2 3 4 5 6 7 8 9 10 都是

1. 能与家人或朋友沟通交流

从来
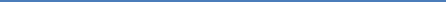
 一直

没有 0 1 2 3 4 5 6 7 8 9 10 都是

1. 得到医生和护士的支持

从来
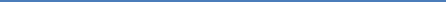
 一直

没有 0 1 2 3 4 5 6 7 8 9 10 都是

1. 可以恢复工作或做寻常家务

从来
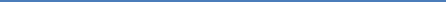
 一直

没有 0 1 2 3 4 5 6 7 8 9 10 都是

1. 感觉舒适、能控制情绪

从来
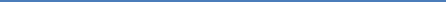
 一直

没有 0 1 2 3 4 5 6 7 8 9 10 都是

1. 总体上感觉好转

从来
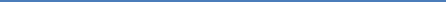
 一直

没有 0 1 2 3 4 5 6 7 8 9 10 都是

**B部分**

您在过去的24小时里有以下情况吗？

（选择0到10, 10表示从来没有[很好]，0表示一直都是[极差]）

1. 中度疼痛 从来
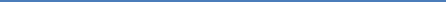
 一直

没有 10 9 8 7 6 5 4 3 2 1 0 都是

1. 剧烈疼痛 从来
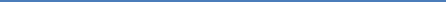
 一直

没有 10 9 8 7 6 5 4 3 2 1 0 都是

13.恶心呕吐 从来
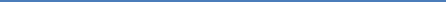
 一直

没有 10 9 8 7 6 5 4 3 2 1 0 都是

14.感觉担心或焦虑 从来
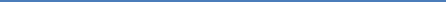
 一直

没有 10 9 8 7 6 5 4 3 2 1 0 都是

15.感觉悲伤或低落 从来
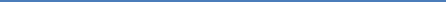
 一直

没有 10 9 8 7 6 5 4 3 2 1 0 都是

**Supplementary Table 1**. Multivariable Linear Regression Analyses of the Intervention on Primary and Secondary Outcomes

| Variable & Model | Unstandardized Coefficients B (95% CI) | P value | R^2^ | Adjusted R² | F value |
| --- | --- | --- | --- | --- | --- |
| **Model 1: dependent variable - POD3 QoR-15 score** | | | | | |
| Constant | 115.17 (113.92, 116.42) | ＜0.001 | 0.284 | 0.273 | 27.131 |
| **Group**​ (K group vs. C group) | 5.52 (3.97, 7.06) | ＜0.001 |  |  |  |
| Norepinephrine Use (yes vs. no) | 0.00 (-1.75, 1.75) | 0.999 |  |  |  |
| **Model 2: dependent variable - Dosage of sufentanil (ug)** | | | | | |
| Constant | 38.05 (36.08, 40.02) | ＜0.001 | 0.461 | 0.453 | 58.512 |
| **Group​** (K group vs. C group) | -12.61 (-15.02, -10.19) | ＜0.001 |  |  |  |
| Norepinephrine Use (yes vs. no) | 0.23 (-2.50, 2.97) | 0.867 |  |  |  |
| **Model 3: dependent variable - Dosage of remifentanil (mg)** | | | | | |
| Constant | 2.58 (2.42, 2.74) | <0.001 | 0.102 | 0.089 | 7.755 |
| **Group**​ (K group vs. C group) | -0.39 (-0.58, -0.19) | <0.001 |  |  |  |
| Norepinephrine Use (yes vs. no) | -0.03 (-0.25, 0.19) | 0.794 |  |  |  |
| **Model 4: dependent variable - CV-MAP** | | | | | |
| Constant | 0.147 (0.133, 0.161) | <0.001 | 0.041 | 0.027 | 2.916 |
| **Group**​ (K group vs. C group) | 0.018 (0.001, 0.034) | 0.033 |  |  |  |
| Norepinephrine Use (yes vs. no) | -0.004 (-0.022, 0.015) | 0.703 |  |  |  |
| **Model 5: dependent variable - CV-HR** | | | | | |
| Constant | 0.151 (0.139, 0.163) | <0.001 | 0.096 | 0.083 | 7.260 |
| **Group**​ (K group vs. C group) | -0.027 (-0.042, -0.012) | <0.001 |  |  |  |
| Norepinephrine Use (yes vs. no) | 0.003 (-0.015, 0.020) | 0.757 |  |  |  |

CV-MAP: Coefficient of variation of mean arterial pressure; CV-HR: Coefficient of variation of mean heart rate.
